# Nurse-surgeons in the Australian public health system: A descriptive quantitative survey

**DOI:** 10.1016/j.ijnsa.2024.100268

**Published:** 2024-11-19

**Authors:** Tenber Grota, Adam Burston, Vasiliki Betihavas, Elisabeth Jacob

**Affiliations:** a School of Nursing, Midwifery and Paramedicine, Australian Catholic University, Sydney, NSW 2060 Australia; b Nursing Research and Practice Development Centre, The Prince Charles Hospital, Chermside, QLD 4032 Australia; c School of Nursing & Midwifery, University of Notre Dame Australia, 160 Oxford St Darlinghurst NSW 2010 Australia

**Keywords:** Advanced practice nursing, Nurse practitioners, Nurse-surgeon, Surgical procedures, Operative, Surveys and questionnaires, Universal health care, Waiting lists

## Abstract

**Background:**

With over five billion people worldwide lacking access to surgery, innovative solutions are vital to address the global surgical crisis. Nurse-surgeons present a promising innovation. Considering their contribution worldwide and impact on surgical care in Australia, an exploration of these advanced practice nurses is timely.

**Objective:**

To investigate the roles, training, education, and perceptions of career prospects and support received by practicing nurse-surgeons in the Australian public health system.

**Design:**

Non-experimental descriptive national survey

**Methods:**

The target population was nurse-surgeons practicing within the Australian public health system. The survey questionnaire comprised of four sections containing questions on nurse-surgeon demographics, roles, training, and perceptions of career prospects and support received. Data collection was conducted through emailing of public hospitals, crowdsourcing, and snowballing. Descriptive analysis was used to report the findings.

**Results:**

Twenty-eight nurse-surgeons participated in the study, 22 females and six males. Most commonly, participants (*n* = 10) held master's degrees and trained to become nurse-surgeons for an average of 2·27 years (95 % CI [1·47,3·07]). Training programs varied but were all surgical specialty-specific, and usually included a practical component, theoretical component, and competency assessment prior to independent practice. Participants rated employment prospects for nurse-surgeons as poor to average due to limited work opportunities, politics, and strong pushbacks from Australian medical societies. The support received from nurses, surgeons and management was rated by participants as good providing reasons such as supportiveness, value recognition, jealousy, and resentment. The participants were very likely (95 % CI [7.436 – 9.364] to continue practicing due to positive job satisfaction but recommended the standardising of training and practice to ensure role futureproofing.

**Conclusions:**

Nurse-surgeons have been practicing in Australia for decades, yet no standard training and credentialing pathway exist for them. This study identified the various roles, non-standard training, and perceptions of nurse-surgeons in the Australian public health system. The findings of this study will have an impact on policymakers and stakeholders to develop standard national credentialing pathway for nurse-surgeons in Australia to enhance clinical practices and ensure a consistent framework for recognition and development of these advanced practice nurses.

## Abstract (author highlights)

• Modern nurse-surgeons have been practicing globally since the 1950s.
• Nurse-surgeon implementation in many local systems around the world has led to surgical waitlist reduction, timely patient access to essential surgeries in remote and rural communities, and prevention of mortalities.
• Nurse-surgeon practice in Australia may have started in the late 1970s to early 1980s.
• This study found Australian nurse-surgeon roles spanning across diverse specialties such as endoscopic and general surgeries and engaging in supplementary roles, including research and nurse-led clinic consultations.
• There is a need for a standard national credentialing pathway for nurse-surgeons in Australia.

**What is already known?**

Modern nurse-surgeons have been practicing globally since the 1950s.

Nurse-surgeon implementation in many local systems around the world has led to surgical waitlist reduction, timely patient access to essential surgeries in remote and rural communities, and prevention of mortalities.

Alt-text: Unlabelled box

**What this paper adds**

Nurse-surgeon practice in Australia may have started in the late 1970s to early 1980s.

This study found Australian nurse-surgeon roles spanning across diverse specialties such as endoscopic and general surgeries and engaging in supplementary roles, including research and nurse-led clinic consultations.

There is a need for a standard national credentialing pathway for nurse-surgeons in Australia.

Alt-text: Unlabelled box

## 1 Background

Surgery, as defined by the World Health Organization, refers to any invasive procedures involving major, minor, open, and minimally invasive laparoscopic and endoscopic techniques performed by surgical providers such as physicians, nurses, and other healthcare professionals to diagnose and or treat surgical conditions (Debas et al., 2006). Millions of people undergo surgery every year which is oftentimes their last resort to avoid disease progression or death (World Health Organization, 2022). Access to surgery is a fundamental element of universal health care (World Health Organization, 2014). However, surgery continues to be widely disregarded as a health agenda of global concern even with data suggesting that 62% of the world's population or at least five billion people have no access to surgery (Alkire et al., 2015; Chamie, 2020; Meara et al., 2015) resulting in more than 18 million preventable deaths annually from surgically treatable conditions (Reddy et al., 2020).

Limited access to surgery disproportionately affects developing countries, highlighting global inequities in surgical care (Bath et al., 2019). This disparity extends to countries without universal health care (Reddy et al., 2020). The COVID-19 pandemic strained even advanced health systems, exacerbating surgical challenges (Muralidar et al., 2020; Wiersinga et al., 2020). Ongoing restrictions and lockdowns amplified the impact, leading to a surge in elective surgery cancellations and worsening pre-existing surgical waiting lists (Carr et al., 2021; COVIDSurg Collaborative, 2020).

The factors that affect timely patient access to surgery are numerous. Regardless, the billions of people globally that require surgery, and the debilitating clinical outcomes arising from surgical delays, imply that surgical care be considered a priority global health issue and therefore, innovation in surgical care delivery should be upheld to alleviate the global surgical burden. One promising innovation in surgical care provision is the emergence of nurse-surgeons or nurses who undertake surgeries independently (Grota et al., 2022). Despite possessing expert knowledge and clinical competencies for surgery, some nurse-surgeons lack master's degrees - a recommendation by the International Council of Nurses (2008) to be considered an advanced practice role. Therefore, as a group, nurse-surgeons do not fit these criteria. Nurse-surgeon titles and trainings are unregulated and are usually based on their area of surgical specialty which include nurse endoscopist, nurse cystoscopist, nurse hysteroscopist, physican extender, nurse practitioner, surgical care practitioner, clinical nurse specialist, and perioperative specialist practitioner (Grota et al., 2021).

Nurse-surgeons emerged from supply and demand whereby the demand for surgeries is extremely high, but the supply of trained surgeons is critically low (Grota et al., 2022). In developing countries where timely patient access to surgery is dire, the World Health Organization's (2008) Task Shifting strategy extended the scope of practice of differently educated healthcare providers to perform complex clinical duties that used to be performed by clinicians historically assigned those tasks. Task shifting may have been pivotal in recognising the value of nurse-surgeons even though modern nurse-surgeons have been documented to be performing surgeries such as c-sections and laparotomies since the 1950s (White et al., 1987). Similarly, in developed countries such as Australia, surgical capacities needed to be innovated to meet the growing surgical demands amidst the ageing medical workforce (OECD iLibrary, 2019), junior physicians being restricted from working unsafe hours (Philibert et al., 2002), the longstanding uneven geographical distribution of surgeons (Phillips, 2022), and specialist surgeons managing multimorbid patients who are now living longer than average due to modern technology and healthcare (Vivekanantham & Gnanappiragasam, 2014).

The earliest known use of the term “nurse-surgeon” dates back to the 1500s when King Henry VIII named William Bullein his private nurse-surgeon (Duffin, 2017). However, the earliest known document that described “nurse-surgeons” as nurses capable of independently performing surgeries was published in the 1980s when due to the shortage of surgeons and the demand for emergency obstetric and gynaecological surgeries, a need to train a group of African nurse-surgeons to perform surgery arose (White et al., 1987). Physicians and nurses campaigned for the standardisation of nurse-surgeons throughout the 1980s (Judy 1985; Litt & Brodsky, 1983). However, this momentum declined in the 1990s until publications about nurse-surgeons resurfaced in the 2000s (Kingsnorth, 2005; Kowalewski & Jahn, 2001; Marsh, 2005; Zorn, 2005) where nurse-surgeons were recognised as qualified non-physician surgeons in the United Kingdom (Mickute, 2009). Nowadays, nurse-surgeons practice different types of surgeries such as general, plastics, orthopaedics, cardiovascular, obstetrics, gynaecology, and orotolaryngology in Europe, Africa, Asia, and North America (Grota et al., 2021; Kowalewski & Jahn, 2001; Zorn, 2005). Some of these surgeries include (but are not limited to) caesarean section, laparotomy, hysterectomy, appendicectomy, herniorrhaphy, endoscopy, hysteroscopy, cystoscopy, biopsy, and carpal tunnel release (Grota et al., 2021).

The impact of nurse-surgeons in global surgical care delivery has been crucial in saving at-risk women who are pregnant and require emergency caesarean sections and hysterectomies, patients with life-threatening blood clots requiring angiograms and percutaneous thrombectomies, and other patients requiring essential gynaecologic, endoscopic, and urological surgeries in remote and urban settings (Bodle et al., 2008; Dryer, 2006; Gidlow et al., 2000; Redwood, 2009; White et al., 1987; Wright, 2000). Nurse-surgeon implementation in many parts of the world has led to surgical waitlist reduction (Grota et al., 2022), timely patient access to essential surgeries both in remote and rural communities (Johal & Dodd, 2017; Redwood, 2009) and prevention of mortalities that could have been added to the 18 million preventable surgical access-related deaths (Reddy et al., 2020).

In Australia, the earliest documented use of nurse-surgeons was in 2004, when a group of South Australian nurse-surgeons performed endoscopies in recognition of the goal of the National Bowel Screening Program of the Australian government to prevent deaths of Australians from bowel cancer, a type of cancer that is easily treatable if detected early. Bull et al. (2006) reported a 96.2% success rate of nurse-surgeon performed endoscopies without any documented mortalities, complications, and serious adverse events. Beck (2013), Duncan et al. (2017), and Cusack et al. (2018) conducted their own studies of nurse-surgeons undertaking endoscopies and concluded similar positive outcomes.

In 2012, Sapre et al. (2012) explored the potential of nurse-surgeons in easing the burden of delivering urological surgical services while aiding in decreasing the workload of urologists. The study reported a reduction in the flexible cystoscopy waitlist by 65%. The five studies (Cusack et al., 2018; Duncan et al., 2017; Beck, 2013; Sapre et al., 2012; Bull et al., 2006) on nurse-surgeons are currently the only known published literature on nurse-surgeons in Australia. However, taking into account the international data on nurse-surgeons, it can be assumed that there might be other nurse-surgeons practicing in Australia yet to be studied.

Given the significant role of nurse-surgeons globally and their potential to enhance surgical services in Australia, this study addresses the absence of an Australian quantitative research on nurse-surgeons. The lack of standardised nursing titles, highlighted by Duffield et al. (2011), poses challenges, necessitating the use of the term “nurse-surgeon” as an overarching designation. Standardising this language is crucial for accurate data consolidation. The study explores the roles, training, education, and perceptions of career prospects and support received by nurse-surgeons within the Australian public health system.

## 2 Methods

This study is the first phase of a two-phased research project exploring currently practicing nurse-surgeons in Australia.

### 2.1 Aim

To investigate the roles, training, education, and perceptions of career prospects and support received by practicing nurse-surgeons in the Australian public health system.

### 2.2 Design

This study utilised a cost-effective non-experimental descriptive survey design using REDCap® (2022) for data collection among geographically dispersed nurse-surgeons. The cross-sectional survey, designed with input from surgical and perioperative nurses, covered demographics, roles, training, and perceptions. The instrument underwent expert validation and pre-testing for usability, ensuring robust data collection (Prince, 2012). The participant information letter (see Supplementary material 1) was included in page one of the survey. The final survey instrument details are available in Supplementary Material 2.

### 2.3 Inclusion and/or Exclusion Criteria

The participants of this study were currently practicing nurse-surgeons within the Australian public health system with active Registered Nurse or Nurse Practitioner Australian Health Practitioner Regulation Agency registration. Due to time and cost constraints in data collection of a nationwide survey, only those working in public hospitals recognised by the Australian Department of Health (2022) were included. All surgical specialties and settings were considered. Nurse surgical assistants such as Perioperative Nurse Surgeon's Assistant, Registered Nurse First Assistant, Registered Nurse First Surgical Assistant, and Non-Medical Surgical Assistant were excluded as they do not perform surgeries independently, only under the direct instruction and supervision of a surgeon or nurse-surgeon.

### 2.4 Sampling

As there is no known official record that clearly defines the classification of nurse-surgeons in Australia, it was difficult to ascertain the exact sample size for this study. Therefore, no sampling frame was determined, and convenience sampling was used to reach as many participants as possible based on their ease of accessibility.

### 2.5 Data collection

To maximize recruitment of hard-to-reach nurse-surgeon participants and address potential bias, three strategies were employed (Palmer & Strickland, 2016; Parker et al., 2019). Public hospitals across Australia were contacted in stages, and those responding were sent the survey link to distribute, ensuring participant anonymity. Crowdsourcing involved the first author presenting at the 2022 Australian College of Perioperative Nurses’ International Conference, where a Quick Response code facilitated convenient and cost-effective recruitment. Snowballing, suitable for elusive subjects like nurse-surgeons, utilised the authors' professional LinkedIn®, ResearchGate® and Facebook® accounts and involved seeding nursing groups, such as the Australian College of Perioperative Nurses and the Australian College of Nurse Practitioners, to disseminate the survey link through various channels such as electronic direct mail, e-magazine and as a post in the research section of their website.

#### 2.5.1 Emailing of public hospitals

**Fig. 1:**
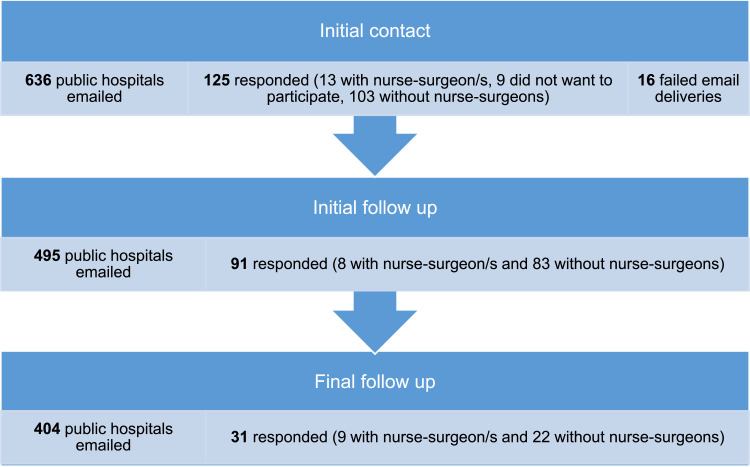
Number of respondents at initial contact, initial follow up and final follow up.

The initial contact stage, starting in March 2022, involved reaching out via email to 636 public hospitals out of the 680 recognised by the Department of Health (2022). Subsequent follow up emails were sent in April and June to non-responding hospitals. Overall, 247 (39%) of the 620 public hospitals responded, with 30 confirming nurse-surgeon/s, 208 reporting none, and nine declining to participate. Fig. 1 illustrates the respondent flow at initial contact, initial follow-up, and final follow-up.

#### 2.5.2 Crowdsourcing and snowballing

The snowballing recruitment commenced in June 2022 while the crowdsourcing event occurred in one day on 23 July 2022. It was impossible to trace the number of respondents during the crowdsourcing event and the snowballing recruitment as the survey was designed to ensure anonymity of the participants.

### 2.6 Data analysis

Descriptive statistics was used to report the findings. No statistical software was used to analyse the collected data. As a cross-sectional survey, loss to follow up was not addressed. No modification of variables occurred during data analysis. Data that were *missing completely at random* or *missing at random* were accepted and left as is. Imputation of missing data was not conducted as the study was an explorative survey of a limited number of study subjects who have diverse characteristics and to the authors’ best knowledge, have never been studied as one population. Therefore, the data cannot be imputed without marring the integrity of the collected data. As inferential statistical analysis was not used, sensitivity analysis was not conducted.

### 2.7 Ethical considerations

The study was approved by the Australian Catholic University Human Research Ethics Committee prior to recruitment, and the study was conducted in accordance with the National Statement on Ethical Conduct in Human Research (National Health and Medical Research Council, 2018). All participants provided consent to participate prior to accessing and completing the survey. Survey responses were encrypted and stored in a secure cloud-based repository. Personal identifiers were removed as soon as data were collected, and only aggregated data were reported.

## 3 Results

### 3.1 Characteristics of the study participants

A total of 38 unique visitors clicked the survey link. Thirty-three consented to complete the survey while five did not consent. Of the 33 that consented, five did not continue and left all the succeeding fields blank. The data sets of these five visitors were removed leaving 28 study participants who completed or partially completed the survey. As all the missing data were *missing completely at random*, missingness was handled by acceptance and any missing data were left as is. As this study was exploratory, no response rate was calculated.

### 3.2 Nurse-surgeon demographics

The demographics of the study participants are shown in Table 1. Twenty-two of the participants were females and six were males. The most common age range was 35 to 44 years (n = 12). The state where most of the participants practiced was Victoria (n = 14) followed by Queensland (n = 6). None practiced in the Australian Capital Territory and Tasmania. Most participants were working permanent part time hours (n = 15) followed by permanent full timers (n = 9), self-employed (n = 3) and one casual employee.

**Table 1:** Nurse-surgeon demographics.

	**n**	**%**
**Sex**		
Female	22	78.6
Male	6	21.4
**Total**	**28**	**100**
**Age group**		
65 and older	1	3.6
55-64	5	17.9
45-54	7	25.0
35-44	12	42.9
25-34	2	7.1
18-24	1	3.6
**Total**	**28**	**100**
**Employment status**		
Casual	1	3.6
Permanent full time	9	32.1
Permanent part time	15	53.6
Self-employed	3	10.7
**Total**	**28**	**100**

### 3.3 Nurse-surgeon roles

Participants were practicing as Registered Nurses (n = 16), Nurse Practitioners (n = 6) and those with both Registered Nurse and Nurse Practitioner registrations (n = 5). Predominant clinical settings included the operating theatre or perioperative department (n = 14), endoscopy unit (n = 13), day surgery unit (n = 12), and hospital outpatient clinic (n = 8). Ten participants had over a decade of nurse-surgeon experience, with a mean of 18.13 years for those with 10 or more years (95% CI [7.48, 28.77]). Surgical specialties encompassed endoscopy (n = 10), general surgery (n = 6), urology (n = 6), gynaecology (n = 4), plastic surgery (n = 4), cardiac (n = 3), and obstetrics (n = 3). Common endoscopic surgeries included colonoscopy (n = 11), flexible sigmoidoscopy (n = 10), gastroscopy (n = 6), flexible cystoscopy (n = 4), endoscopic vein harvesting (n = 3) and rigid cystoscopy (n = 3). Non-endoscopic nurse-surgeon performed surgeries included biopsy across different specialties (n = 8), open vein harvesting (n = 3), circumcision (n = 1), carpal tunnel release (n = 1), and inguinal hernia repair (n = 1). Beyond nurse-surgeon roles, the majority (n = 19) engaged in additional work like clinical nurse consulting, outpatient clinic appointments, and surgical assisting. The roles of the study participants are detailed in Table 2.

**Table 2:** Nurse-surgeon roles grouped by geographical area of practice.

	**Metropolitan**		**Regional**		**Total**	
	n	%	n	%	n	%
**Current Ahpra registration** Registered Nurse Nurse Practitioner Both Other Total	11 4 3 0	61.1 22.2 16.7 0.0	5 1 3 1*	50.0 10.0 30.0 10.0		
	18	100	10	100	28	100
**Clinical practice setting** Hospital Community / primary health service Own Nurse Practitioner clinic Total	17 1 0	94.4 5.6 0.0	8 0 2	80.0 0.0 20.0		
	18	100	10	100	28	100
**Experience as nurse-surgeon** 10 years or more 7 years but less than 10 years 4 years but less than 7 years 1 year but less than 4 years Less than a year Missing data Total	7 2 3 5 0 1	38.9 11.1 16.7 27.8 0.0 5.5	3 2 2 2 0 1	30.0 20.0 20.0 20.0 0.0 10.0		
	18	100	10	100	28	100
**Previous experience as nurse** 10 years or more 7 years but less than 10 years 4 years but less than 7 years 1 year but less than 4 years Less than a year Missing data Total	8 1 4 1 1 3	44.4 5.6 22.2 5.6 5.6 16.7	3 4 0 1 0 2	30.0 40.0 0.0 10.0 0.0 20.0		
	18	100	10	100	28	100
**Main role before becoming a nurse-surgeon** Theatre nurse / scrub scout nurse Clinical Nurse Consultant Practice nurse Endoscopy nurse Nurse manager Research Missing data Total	5 3 2 2 2 1 3	27.8 16.7 11.1 11.1 11.1 5.6 16.7	5 1 0 2 0 0 2	50.0 10.0 0.0 20.0 0.0 0.0 20.0		
	18	100	10	100	28	100
**Surgical speciality as a nurse-surgeon** Endoscopy General Urology Gynaecology Plastic surgery Cardiothoracic Missing data Total	5 0 4 1 1 3 4	27.8 0.0 22.2 5.6 5.6 16.7 22.2	5 3^#^ - - - 0 2	50.0 30.0 - - - 0.0 20.0		
	18	100	10	100	28	100
**Other roles besides being a nurse-surgeon** Surgical assistant Perioperative nurse Research Nurse Practitioner clinic consultations Did not specify No other roles Missing data Total	1 3 2 2 2 4 4	5.6 16.7 11.1 11.1 11.1 22.2 22.2	1 4 0 3 0 0 2	10.0 40.0 0.0 30.0 0.0 0.0 20.0		
	18	100	10	100	28	100
**Percentage of work as a nurse-surgeon** 100% 75% or less than 100% 50% or less than 75% 25% or less than 50% Less than 25% Missing data Total	2 3 2 6 1 4	11.1 16.7 11.1 33.3 5.6 22.2	0 4 1 1 1 3	0.0 40.0 10.0 10.0 10.0 30.0		
	18	100	10	100	28	100

*Advanced Practice Nurse Endoscopist; **#** combination of general, urology, gynaecology, plastics and orthopaedics

### 3.4 Nurse-surgeon education and training

Participant education and training revealed ten with master's degrees, six with bachelor's degrees, and one holding a Doctor of Philosophy degree. Fourteen underwent postgraduate studies before practicing as nurse-surgeons, while seven did not require postgraduate studies. Twelve participants had a blend of formal and informal practical training, and an equal number had a mix of formal and informal education. During practical training, 15 participants were supervised by surgeons, four by both surgeons and nurse-surgeons, and one by a nurse-surgeon alone. Twenty participants underwent competency assessments before independent practice, with approval decisions made by clinical supervisors (n = 6) or a joint approval from various stakeholders (n = 13). Eight participants reported two or more years of comprehensive nurse-surgeon training, eight reported one to two years, and five reported less than one year. The mean training duration for those specifying years was 2.27 years (95% CI [1.47, 3.07]). The education and training of participants are shown in Table 3.

**Table 3:** Nurse-surgeon education and practical training.

	**n**	**%**
**Education** Master Bachelor Postgraduate diploma Doctor of Philosophy Attended university but did not complete	10 7 2 1 1	47.6 33.3 9.5 4.8 4.8
Total	21	100
**Required to undertake postgraduate study prior to independent practice as a nurse-surgeon** Yes, at university Yes, program organised by the State Yes, but did not specify Yes, in house training not at university No	6 3 3 2 7	28.6 14.3 14.3 9.5 33.3
Total	21	100
**Received practical training prior to independent practice as a nurse-surgeon** Yes, formal training Yes, a combination of formal and informal practical trainings Yes, informal training No	13 4 3 1	61.9 19.0 14.3 4.8
Total	21	100
**Received theoretical teaching prior to independent practice as a nurse-surgeon** Yes, a combination of formal and informal education Yes, formal education Yes, but did not specify Yes, informal education Other	9 5 4 2 1	42.9 23.8 19.0 9.5 4.8
Total	21	100
**Supervisor during practical training and education** Surgeon Both surgeon and nurse-surgeon Nurse-surgeon	15 5 1	71.4 23.8 4.8
Total	21	100
**Required to pass a competency assessment prior to independent practice as a nurse-surgeon** Yes, formal competency assessment Yes, a combination of formal and informal competency assessments Yes, informal competency assessment Yes, but did not specify No	13 4 2 1 1	61.9 19.0 9.5 4.8 4.8
Total	21	100
**Final decision to perform surgeries independently** Surgeon clinical supervisor Joint approval from the surgeon, nurse-surgeon clinical supervisors, and hospital management Joint approval from the surgeon, nurse-surgeon clinical supervisors, hospital management and hospital quality team Joint approval from the surgeon, nurse-surgeon clinical supervisors, hospital management, hospital quality team, and the representative from the public health system of the state Hospital management Joint approval from the surgeon and nurse-surgeon clinical supervisors Joint approval from surgeon clinical supervisor, hospital management, quality team Representative from the public health system of the state Other	6 2 2 2 1 1 1 1 5	28.6 9.5 9.5 9.5 4.8 4.8 4.8 4.8 23.8
Total	21	100
**Length of training (inclusive of practical training, educational preparation and competency assessment)** Two years or more ([n=8] One year but less than two years (n=7] Less than 1 year [n=3] Did not specify [n=3]	8 7 3 3	38.1 33.3 14.3 14.3
Total	21	100

### 3.5 Nurse-surgeon perceptions

Participants' perceptions of career prospects and support during training and practice were assessed on a 5-point scale with 1 being terrible and 5 being excellent (see Table 4). For employment prospects, three participants rated as excellent (rating of 5), eight as good (rating of 4), five as average (rating of 3), five as poor (rating of 2), and four as terrible (rating of 1), resulting in a mean of 2.62 or poor to average (95% CI [2.11, 3.13]). Regarding support during training, ratings were six excellent, five good, five average, four poor, and one terrible, with a mean of 3.52 or good (95% CI [2.95, 4.09]). Support during independent practice received ratings of 11 excellent, seven average, two good, and one terrible, resulting in a mean of 4.05 or good (95% CI [3.52, 4.58]). From surgeons’ support during training, ratings included 13 excellent, four average, two terrible, one good, and one poor, with a mean of 4.05 or good (95% CI [3.41, 4.69]). During independent practice, seven participants rated surgeons’ support as excellent, six as average, five as good, and two as poor, with a mean of 3.85 or good (95% CI [3.36, 4.34]). Management support during training received 10 excellent, four good, four average, and two poor ratings, leading to a mean of 4.1 or good (95% CI [3.6, 4.6]). After training, ratings for management support were seven excellent, six average, five good, and two poor, resulting in a mean of 3.85 or good (95% CI [3.36, 4.34]). Participants' likelihood of continuing as nurse-surgeons was scored on a 10-point likert with 10 being extremely likely and 0 being extremely unlikely. This question had a mean rating of 8.4 or likely (95% CI [7.52, 9.28]), with most participants (n = 10) indicating a likelihood of 10 or extremely likely. Additional participant comments emphasised the need for formal training recognition, challenges in acceptance, and recognition of nurse-surgeons, as well as concerns about governing bodies and political aspects in nursing roles. More detailed comments can be found in Table 4.

**Table 4:** Nurse-surgeon perceptions (ratings: excellent, good, average, poor, terrible).

	Excellent		Good		Average		Poor		Terrible		Total	
	n	%	n	%	n	%	N	%	n	%	N	%
Employment prospects in Australia	1	4.8	3	14.3	8	38.1	5	23.8	4	19.0	21	100
Support received from nursing colleagues at work during training	7	33.3	5	23.8	4	19.0	4	19.0	1	4.8	21	100
Support received from nursing colleagues at work as a practising nurse-surgeon	11	52.4	2	9.5	7	33.3	0	0	1	4.8	21	100
Support received from surgeons at work during training	13	61.9	1	4.8	4	19.0	1	4.8	2	9.5	21	100
Support received from surgeons at work as a practising nurse-surgeon	11	55.0	1	5.0	6	30.0	1	5.0	1	5.0	20	100
Support received from management during training	10	50.0	4	20.0	4	20.0	2	10.0	0	0	20	100
Support received from management as a practising nurse-surgeon	7	35.0	5	25.0	6	30.0	2	10.0	0	0	20	100

## 4 Discussion

### 4.1 Interpretations

The findings of this study indicate that nurse-surgeons exist in a variety of roles within the Australian public health system. One participant with nurse-surgeon experience of 42 years indicates that nurse-surgeon practice in Australia may have started in the late 1970s to early 1980s. Although the first documented nurse-surgeon performed surgeries in the 1950s (White et al., 1987), it was not until the 1980s (Judy 1985; Litt & Brodsky, 1983) when active campaigns to develop nurse-surgeon roles in Western countries proliferated, which was around the same decade when one of the study participants started practicing as a nurse-surgeon in New South Wales.

Most of the study participants had at least 20 years of experience as scrub/scout nurses prior to becoming nurse-surgeons. The majority were master's degree holders who underwent nurse-surgeon training for two years and three months, which was surgical-specialty specific and included practicum, educational preparation, and competency assessment. They were required to pass a competency assessment and an approval from the clinical supervisor and/or external the hospital management, hospital quality team and external credentialing committee prior to independent practice. These findings are similar to an international review (Grota et al., 2021) which found that nurse-surgeons undergo a surgical-specialty specific training program that includes a theoretical component and a practical component that is supervised by surgeons before being assessed for competency to practice independently. The study participants stated the surgeries they performed independently including biopsy, inguinal hernia repair, circumcision, colonoscopy, gastroscopy, cystoscopy, and hysteroscopy. These were surgeries documented to have been performed independently by nurse-surgeons globally (Grota et al., 2022).

Participants rated the employment prospects for nurse-surgeons in Australia as poor to average due to four primary reasons. First, the role is dependent on hospital approvals hence limited mobility and work opportunities for nurse-surgeons exist, as the role is currently highly uncoordinated and hospital specific and may not carry across networks and states. Second, politics within hospitals arise from factors such as nurse-surgeons being seen as taking away training opportunities for junior surgeons and some physicians believing that nurses should not have this role. Third, strong pushbacks from Australian medical societies against implementation of a nurse-surgeon role. Fourth, the inability of nurse-surgeons to access Medicare Benefits Schedule rebates and associated payment issues. Innovation and change can always be clouded by fear of the unknown (Newton, 2019) and arising from this fear is the failure of stakeholders such as physicians and hospital management to recognise the value of such clinical practice innovation. The benefits of nurse-surgeons in improving surgical capacity and the timely delivery of surgical care greatly outweigh and therefore justify challenging these barriers.

The support that the nurse-surgeons received from their nursing colleagues during training and independent practice was good. Although some participants highlighted jealousy or tall poppy syndrome as the reasons why their nursing colleagues refuse to accept the concept of nurses performing surgeries and needing surgical assistance, most were supportive and recognised the value of nurses in advanced practice roles. This also creates career growth opportunities for experienced nurses wanting to expand their career without leaving clinical practice. Similarly, the support that nurse-surgeons received from surgeons was good during training and independent practice. The surgeons who supervised the participants were supportive, encouraging and available for feedback and discussion when required. However, other surgeons who are not involved with their training, have not been exposed to nurse-surgeons, or new to surgery were not as supportive. The hospital management's support on the participants during their training and independent practice was also good. This was evidenced by the continually supportive management and the appreciation of the value nurse-surgeons add to theatre capacity. However, as with their nursing colleagues, the participants believed that management lacked the insight to fully understand the role leading to payment issues. Support was subjective as seen in the contradicting participant responses even though the ratings were the same. Disputes and resistance in professional role boundaries in the health sector are well documented and longstanding particularly when the established or assumed to be protected roles of a specific healthcare professional is being threatened in the course of another healthcare professionals’ scope of practice expansion and evolution (King et al., 2015). This is similar to the plight of Nurse Practitioners in the United States wishing to gain title recognition and standardisation of the scopes of advanced practice receiving continued strong resistance from medical societies (Poghosyan et al., 2018).

With an average of 8.4 (95% CI [7.436 – 9.364] on a scale of 0 to 10, the participants were very likely to continue their nurse-surgeon practice mainly due to job satisfaction. However, as the role is not recognised by medical and nursing disciplines, the participants believed that there are still factors to consider for the futureproofing of the role such as practice standardisation through formal training, fellowship program from the Australian College of Nurse Practitioner, allowing nurse-surgeons to access Medicare Benefit Schedule, and gathering support from governing bodies and nursing unions.

### 4.2 Limitations

This study faces a potential confounder due to the lack of a standard for nurse-surgeon practice in Australia, resulting in blurred scopes of practice with nurse surgical assistants. Despite explicit eligibility criteria, some nurse-surgeons working as non-medical surgical assistants introduced uncontrollable variables, evident in outlier responses like participant ID 20, who claimed to independently perform surgeries typically beyond the documented scope of nurse-surgeons, raising questions about accuracy and adherence to eligibility criteria.

Another limitation of this study is the number of survey responses received which could have been higher if the email addresses of the public hospitals’ appropriate contact person were gathered. The first author searched for email addresses by visiting the websites of each public hospital and many of the email addresses retrieved were generic. An attempt was made to gather the correct email addresses of these public hospitals by contacting the Australian Institute of Health and Welfare but due to cost constraints, this data was not secured.

A final limitation of this study is the uncontrollable and unpredictable responses from the public hospitals during recruitment. For example, a Director of Nursing of one hospital confirmed on the initial contact the existence of nurse-surgeons in their facility. However, when this Director of Nursing consulted with the head of their research department who happened to be a surgeon, the public hospital retracted their statement and stated that no nurse-surgeons exist in the facility. Consequently, practicing nurse surgeons that may have provided useful data may have been missed from the study.

### 4.3 Recommendations for Further Research

The findings of this study present many opportunities for future research. First, this study provides an emerging evidence base to support deeper exploration of the benefits nurse-surgeons provide to healthcare delivery in Australia and overseas. Second, international research on the standardisation of nurse-surgeon practice and their potential in easing the global surgical burden should be undertaken. Third, investigation of methods to confront and negate barriers to the utilisation of nurse-surgeons practice in Australia should be implemented as part of a national strategy to address the ongoing and deteriorating health workforce situation. Finally, a government-supported national database tracking nurse-surgeons and their practice areas would aid future research.

### 4.4 Implications for policy and practice

Stakeholders and policymakers in Australia such as the Department of Health, Australian Health Practitioner Regulation Agency, local health systems, Australian College of Perioperative Nurses, Australian College of Nurse Practitioner, Australian College of Nursing, and Australian Medical Council can use the findings from this study to collaboratively develop a formal and standard national credentialing pathway for nurse-surgeons. Therefore, innovative and difficult conversations addressing the persistent health workforce challenges may need to be initiated with the government, surgical healthcare professionals such as physicians and nurses, and local health systems to develop sustainable and collaborative solutions to enhance the surgical capacity across Australia.

## 5 Conclusion

Nurse-surgeons have existed within the Australian public health system performing surgeries for many decades. This study has unearthed the current roles of nurse-surgeons in surgical care, the non-standard trainings nurse-surgeons encounter prior to independent practice, their perceptions on employment prospects, the support received from nursing colleagues, surgeons and management, and the perceived likelihood of continuing to practice as nurse-surgeons in Australia. More high-quality research is necessary to further understand and help define nurse-surgeons scope of practice. Policymakers must consider the findings of this study to develop a standard national credentialing pathway for nurse-surgeons as part of the broader health workforce strategy.

## Data availability

The research data for this study entitled “Nurse-surgeons in the Australian public health system: A descriptive quantitative survey” is stored at Mendeley Data and accessible at https://doi.org/10.17632/nbzs9tndt6.1

## Funding sources

The corresponding author received funding from the Research Training Program of the Australian government to conduct this research.

## CRediT authorship contribution statement

**Tenber Grota:** Writing – original draft, Visualization, Validation, Methodology, Investigation, Formal analysis, Data curation, Conceptualization. **Adam Burston:** Writing – review & editing, Supervision, Resources. **Vasiliki Betihavas:** Writing – review & editing, Supervision, Resources. **Elisabeth Jacob:** Writing – review & editing, Supervision, Resources.

## Declaration of competing interest

The authors declare that they have no known competing financial interests or personal relationships that could have appeared to influence the work reported in this paper.

## Appendix Supplementary materials

Image, application 1

Image, application 2
