# Supplementary material for: Nurse-surgeons in the Australian public health system: A descriptive quantitative survey
Source: Int J Nurs Stud Adv. 2024 Nov 19;7:100268. doi: 10.1016/j.ijnsa.2024.100268 (PMC11612453; doi:10.1016/j.ijnsa.2024.100268)
Supplement: Supplementary file 2 [file mmc2.docx]

**Supplementary material 1**

Survey questionnaire

**Nurse-surgeons in the Australian public health system: Mixed methods study**

You are invited to participate in our national survey of the roles, training, education, and career prospects of nurse-surgeons in the Australian public health system. Nurse-surgeons are nurses trained to perform surgeries independently. For the purpose of this survey, we have adapted the World Health Organisation definition of surgery as any invasive procedures that is performed aseptically, and usually with the use of appropriate anaesthesia, by trained surgeons, other physicians, nurses, and other non-physicians to investigate and/or treat surgical conditions. Please note that Surgical Assistant roles are not considered nurse-surgeons as they do not perform surgeries independently. This survey will take approximately 15 minutes to complete. Your personal information and responses will be anonymous and the data from this research will be reported only in the aggregate. A "Participant Information Letter" is attached below for a comprehensive explanation of this study. Please contact Mr. Tenber Grota on tenber.grota@myacu.edu.au if you have any questions about the survey. Thank you for your time and support.

Please click "I consent" to confirm that you have fully read the "Participant Information Letter" attached below and you are providing your consent to participate in this survey.

**Demographics**

What is your sex?

• Male

• Female

• Other (please specify below)

What is your age?

• 18 – 24

• 25 – 34

• 35 – 44

• 45 – 54

• 55 – 64

• 65 or older

Which state are you currently residing and practicing?

• Australian Capital Territory

• New South Wales

• Northern Territory

• Queensland

• South Australia

• Tasmania

• Victoria

• Western Australia

What is your current employment status?

• Permanent full time

• Permanent part time (please specify below)

• Casual

• Agency

• Self-employed

**Nurse-surgeon roles**

The next set of questions will be about your role as a practicing nurse-surgeon. This information will help us understand your practice setting and duration, surgical specialty, and the surgeries you have performed independently. When answering the questions, please consider the time when you have actually started performing surgeries independently (excluding surgical assisting and traditional perioperative nursing roles).

What is your current Ahpra registration?

• Registered Nurse

• Nurse Practitioner

• Both Registered Nurse and Nurse Practitioner Other (please specify below)

What geographical area are you currently practicing in?

• Metropolitan

• Regional

• Remote

What clinical setting do you currently work in? Select all that apply.

• Operating theatre / perioperative department

• Day surgery unit

• Outpatient clinic (hospital)

• Outpatient clinic (standalone / independent practice)

• Interventional radiology / catheterisation laboratory unit

• Endoscopy unit

• Intensive care unit

• Emergency department

• Community / primary health service

• Other (please specify below)

How long have you been practicing as a nurse-surgeon?

• less than a year

• 1 year but less than 4 years

• 4 years but less than 7 years

• 7 years but less than 10 years

• 10 years or more (please specify below)

How long did you practice as a nurse before becoming a nurse-surgeon?

• less than a year

• 1 year but less than 4 years

• 4 years but less than 7 years

• 7 years but less than 10 years

• 10 years or more (please specify below)

What was your main role before becoming a nurse-surgeon?

• Theatre nurse / scrub scout nurse

• Endoscopy nurse

• Practice nurse

• Emergency nurse

• Critical care nurse

• Nurse manager

• Other (please specify below)

What is your surgical speciality as a nurse-surgeon? Select all that apply.

• Endoscopy

• General surgery

• Gynaecology

• Interventional radiology

• Obstetrics

• Ophthalmology

• Plastic surgery

• Urology

• Other (please specify)

Below are surgeries that are known to have been performed independently by nurse-surgeons worldwide. Which of these surgeries have you performed independently? Select all that apply.

• Angiogram

• Biopsy

• Caesarean section

• Carpal tunnel release

• Circumcision

• Colonoscopy

• Flexible sigmoidoscopy

• Gastroscopy

• Hysterectomy

• Hysteroscopy

• Inguinal hernia repair

• Intravitreal therapy

• Laparotomy

• Percutaneous thrombectomy

• Rigid cystoscopy

• Trauma surgeries (please specify below)

• Other (please specify below)

Do you have other roles at your workplace besides Yes (please specify below) being a nurse-surgeon?

• Yes (please specify below)

• No

What percentage of your work is allocated to being a nurse-surgeon?____________

**Nurse-surgeon training**

The next set of questions will be about your practical training and educational preparation to become a nurse-surgeon. This information will help us understand the specific components of your practical training and the studies you undertook to practice as a nurse-surgeon.

What is the highest academic degree that you have completed?

• Bachelor's degree (please specify below)

• Associate degree (please specify below)

• Attended university but did not complete Master's degree, (please specify below)

• Doctorate or professional degree (please specify below)

• Other (please specify below)

Were you required to undertake postgraduate study before you were allowed to practice as a

nurse-surgeon?

• Yes (please specify below)

• No

Did you receive any practical training before you were allowed to practice independently as a nurse-surgeon?

• Yes - formal training (please specify below)

• Yes - informal training (please specify below)

• Yes - a combination of formal and informal practical trainings (please specify below)

• No practical training

• Other (please specify below)

Did you receive any theoretical teaching or foundation before you were allowed to practice independently as a nurse-surgeon?

• Yes - formal education (please specify below)

• Yes - informal education (please specify below)

• Yes - a combination of formal and informal education (please specify below)

• No education

• Other (please specify below)

Who was your supervisor during your nurse-surgeon practical training and education?

• Surgeon

• Nurse-surgeon

• Both surgeon and nurse-surgeon

• Other (please specify below)

Were you required to pass a competency assessment before you were allowed to practice independently as nurse-surgeon?

• Yes - formal competency assessment (please specify below)

• Yes - informal competency assessment (please specify below)

• Yes - a combination of formal and informal competency assessments (please specify below)

• No competency assessment

• Other (please specify below)

Following competency assessment, who made the final regarding your competence to perform surgeries independently?

• Surgeon clinical supervisor

• Nurse-surgeon clinical supervisor

• Hospital management Hospital quality team

• Representative from the public health system of the state

• Joint approval from the surgeon and nurse-surgeon clinical supervisors

• Joint approval from the surgeon, nurse-surgeon clinical supervisors, and hospital management

• Joint approval from the surgeon, nurse-surgeon clinical supervisors, hospital management, and hospital quality team

• Joint approval from the surgeon, nurse-surgeon clinical supervisors, hospital management, hospital quality team, and the representative from the public health system of the state

• Other (please specify below)

How long was your nurse-surgeon training (inclusive of practical training, educational preparation and

competency assessment)?

• Less than 1 year, please specify (please specify any below)

• One year but less than two years (please specify below)

• Two years or more (please specify below)

**Nurse-surgeon perceptions**

The next set of questions will be about your perceptions as a practicing nurse-surgeon in Australia in terms of career prospects, support received from stakeholders, and the likelihood of continuing your practice.

How would you rate the employment prospects for nurse-surgeons in Australia? Please provide a brief reason for your choice.

• Excellent

• Good

• Average

• Poor

• Terrible

How would you rate the support you received from your nursing colleagues at work during training as a nurse-surgeon? Please provide a brief reason for your choice.

• Excellent

• Good

• Average

• Poor

• Terrible

How would you rate the support you received from your nursing colleagues at work as a practicing nurse-surgeon? Please provide a brief reason for your choice.

• Excellent

• Good

• Average

• Poor

• Terrible

How would you rate the support you received from surgeons at work during training as a nurse-surgeon?

Please provide a brief reason for your choice.

• Excellent

• Good

• Average

• Poor

• Terrible

How would you rate the support you received from surgeons at work as a practicing nurse-surgeon? Please provide a brief reason for your choice.

• Excellent

• Good

• Average

• Poor

• Terrible

How would you rate the support you received from management during training as a nurse-surgeon? Please provide a brief reason for your choice.

• Excellent

• Good

• Average

• Poor

• Terrible

How would you rate the support you received from management as a practicing nurse-surgeon? Pleas provide a brief reason for your choice.

• Excellent

• Good

• Average

• Poor

• Terrible

How likely are you to continue practicing as a nurse-surgeon? Please provide a brief reason for your

choice.

• 0 Extremely unlikely

• 1

• 2

• 3

• 4

• 5

• 6

• 7

• 8

• 9

• 10 Extremely likely

Please provide any additional comments about the nurse-surgeons in the Australian public health system.

When you're finished, please click "Submit" to complete the survey. Clicking "Submit" indicates consent and the inability to withdraw the data due to the anonymous nature of the research.

After clicking "Submit", a separate page will appear which will ask about your interest in participating in the second phase of our study. The invitation page is separate to ensure the anonymity of your responses in this survey.
